# Peptidylarginine Deiminases: An Overview of Recent Advances in Citrullination Research

**DOI:** 10.3390/ijms262412060

**Published:** 2025-12-15

**Authors:** Magdalena Kijak-Boćkowska, Joanna Czerwińska, Agnieszka Owczarczyk-Saczonek

**Affiliations:** 1 Department of Pathomorphology, Maritime Hospital, Gdynia, 81-519 Gdynia, Poland; magdakijak94@gmail.com; 2 Department of Dermatology, Sexually Transmitted Diseases and Clinical Immunology, School of Medicine, Collegium Medicum, University of Warmia and Mazury in Olsztyn, 10-719 Olsztyn, Poland; agnieszka.owczarczyk@uwm.edu.pl

**Keywords:** peptidyl arginine deiminases, citrullination, PAD inhibitors, CECN, autoimmune diseases, cancer, PPAD, NETs

## Abstract

The peptidylarginine deiminase (PAD) family includes five isozymes (PAD1–4 and PAD6) with unique tissue distributions and substrate specificities. These enzymes facilitate citrullination, a post-translational modification where positively charged arginine residues are converted into neutral citrulline residues in the presence of calcium ions. This process significantly changes protein properties, affecting molecular interactions, structural stability, and biological functions. Over the past six years (2019–2025), there has been significant progress in understanding PAD activity mechanisms and their therapeutic potential. Recent discoveries include the regulated nuclear translocation of PAD2, PAD4’s specific role in forming cancer extracellular chromatin networks (CECNs), and the development of next-generation inhibitors with greatly improved pharmacological profiles. PAD4 is crucial in forming neutrophil extracellular traps (NETs). Citrullination of histones H3 and H4 by PAD4 destabilizes chromatin, helping release DNA-protein networks as an antibacterial defense. However, excessive NET formation can contribute to autoimmune diseases and thrombosis. Similarly, the bacterial peptidylarginine deiminase from Porphyromonas gingivalis (PPAD)—the only known prokaryotic citrullinating enzyme—plays a key role. Working with R-gingipains, PPAD triggers pathological citrullination of host proteins, leading to immune tolerance breakdown and linking periodontal disease with systemic autoimmune disorders such as rheumatoid arthritis, atherosclerosis, and Alzheimer’s disease. Once thought to be a rare post-translational modification, citrullination is now understood as a vital regulatory mechanism in both normal physiology and disease, involving both internal processes of homeostasis and external mechanisms of bacterial pathogenesis.

## 1. Introduction

The discovery of citrulline in the structure of hair proteins in 1958 by Professor George Ernest Rogers sparked research into the enzymes responsible for this unusual post-translational modification [1]. Twenty years later, the responsible enzymes were identified, initially named arginine deiminases [2], and then, after it was recognized that they act on arginine residues in peptides rather than on free arginine, they were renamed peptidyl arginine deiminases (PAD) [2,3].

**Figure 1:**
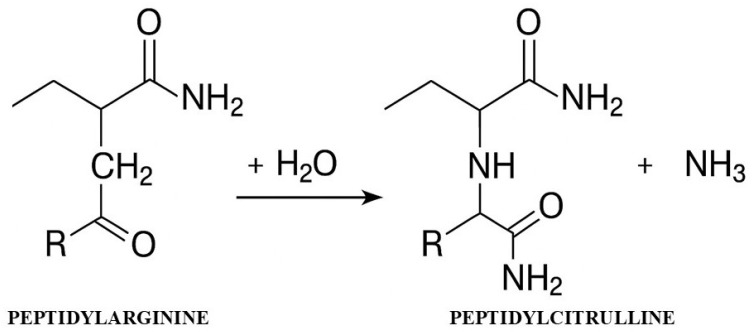
Mechanism of the citrullination reaction catalyzed by PAD enzymes.

Citrullination is an irreversible post-translational modification in which PAD enzymes convert positively charged arginine residues (pArg) into neutral citrulline residues (pCit), resulting in a molar mass change of 1 Da and the release of ammonia [4,5]. This seemingly minor chemical change has enormous biological consequences—the loss of a positive charge drastically alters the electrostatic properties of proteins, affecting their conformation, stability, and ability to interact with other molecules [6] (Figure 1).

The diagram shows the conversion of the arginine residue to citrulline with the participation of the PAD enzyme in the presence of Ca^2+^ ions, leading to the loss of positive charge and the release of ammonia.

## 2. Molecular Architecture and Regulatory Mechanisms

### 2.1. Structure and Domain Organization

PAD enzymes are proteins consisting of 660–665 amino acid residues with a molecular weight of 74–77 kDa [7]. Crystallographic studies have revealed a conservative two-domain architecture, consisting of an N-terminal domain responsible for stabilization and calcium binding, and a C-terminal domain that contains the active site [8,9]. The catalytic center is characterized by an amino acid triad: cysteine (Cys645), histidine (His471), and aspartic acid (Asp473) in the case of PAD4 [10].

#### Differences in Nuclear Organization Between Isoenzymes

PAD4 stands out as the only isoform with a classic nuclear localization signal (NLS: 56-PPAKKKST-63), enabling constitutive translocation to the cell nucleus [5,11]. This unique property allows PAD4 to access chromatin and citrullinate histones directly [12].

Despite the absence of a classic NLS, PAD2 can also translocate to the nucleus via a mechanism regulated by calcium signaling [13]. Groundbreaking research from 2019 showed that, in its resting state, PAD2 is sequestered in the cytoplasm by binding to ANXA5 (annexin A5) [14]. An increase in calcium concentration induces conformational changes in PAD2, weakening its interaction with ANXA5 and enabling transport to the nucleus via the Ran protein [15,16].

PAD1 and PAD3 act mainly in the cytoplasm and do not have nuclear translocation mechanisms [17,18].

### 2.2. Calcium-Dependent Regulation

All PAD enzymes are strictly calcium-dependent, containing 5–6 Ca^2+^ binding sites [14,19]. Calcium ion binding occurs cooperatively, inducing conformational changes that open the active site for substrates [8,20]. Under physiological conditions, at low cytoplasmic calcium concentrations (10^−8^–10^−6^ M), PAD enzymes remain inactive [5,21,22]. Activation requires much higher concentrations (10^−4^–10^−3^ M), which are achieved during apoptosis, terminal differentiation, or cell membrane damage [13,23].

## 3. Tissue Distribution and Physiological Functions

### 3.1. PAD1—Guardian of the Epidermal Barrier

PAD1 is expressed in keratinocytes from the basal layer to the stratum corneum [24]. Together with PAD3, it plays a key role in keratinocyte differentiation through the deamination of proteins, including filaggrin and keratins, and is involved in autophagy processes in the granular layer of the epidermis [22,25]. This process eliminates electrostatic interactions, facilitating the formation of the stratum corneum and maintaining the integrity of the epidermal barrier [26,27] (Table 1).

### 3.2. PAD2—Regulator of Plasticity and Immune Response

PAD2 has the widest tissue distribution of all isozymes [21]. In the central nervous system, it regulates the plasticity of the myelin sheath through citrullination of myelin basic protein (MBP) [28,29]. PAD2 dysregulation is associated with demyelinating diseases, including multiple sclerosis [30].

In the immune system, PAD2 performs multiple functions: it participates in the formation of METosis and NETosis, regulates cell pyroptosis through ASC citrullination and caspase-1 activation, and modulates T cell differentiation [21]. Recent discoveries have revealed its key role in the pathogenesis of sepsis—PAD2 deficiency increases survival in mouse models by reducing caspase-1-dependent pyroptosis [21].

### 3.3. PAD3—Architect of Hair and Skin Structure

PAD3 is mainly located in the medulla and inner root sheath of hair follicles [28], where it citrullinates trichohyalin, a key structural protein of hair [29]. Mutations in the PADI3 gene lead to hair formation disorders, including CCCA and uncombable hair syndrome, through abnormal modification of hair follicle structural proteins [31].

### 3.4. PAD4—Epigenetic Regulator and Defense Mediator

PAD4 is the only isoform constitutively present in the cell nucleus due to its classic nuclear localization signal [32]. It is mainly found in hematopoietic cells: neutrophils, eosinophils, and monocytes [33]. Its unique nuclear localization enables the citrullination of histones H3 and H4, which has effects on chromatin organization and transcription regulation [34,35].

Recent studies have also revealed a non-enzymatic function of PAD4—a physical association with NADPH oxidase subunits (NCF1, NCF2) in neutrophils, where it regulates the production of reactive oxygen species and bactericidal activity [34].

#### Molecular Mechanism of Neutrophil Extracellular Trap (NET) Formation

The formation of neutrophil extracellular traps (NETs) is a complex process involving three essential molecular steps. Elucidation of these mechanisms is crucial for the development of targeted therapeutic interventions [36,37].

The initial phase entails the dissolution of the nuclear envelope, a process that is initiated by the phosphorylation of lamin proteins. PKCα has been shown to phosphorylate and cause the disassembly of lamin B, while CDK4/6 has been observed to phosphorylate lamin A/C [36,38]. These phosphorylation processes, rather than proteolytic cleavage by caspase-3, are responsible for the disruption of the nuclear envelope during NETosis [36]. The translocation of these kinases from the cytoplasm to the nucleus requires a functional actin cytoskeleton in the early stage of neutrophil activation [39,40]. Mice lacking CDK4/6 or PKCα exhibit impaired NET formation in vivo [36,38].

The second stage is nuclear chromatin decondensation, achieved by post-translational modifications of histones. PAD4 catalyzes the conversion of positively charged arginine to neutral citrulline in histones, resulting in the loss of positive charges and the disruption of DNA-histone electrostatic interactions, leading to chromatin decondensation [36,37,41,42]. Concurrently, neutrophil elastase contributes to decondensation through the proteolytic cleavage of histones [37,43]. Kinase-dependent disruption of the nuclear envelope is imperative for the entry of these enzymes into the nucleus. Inhibition of PKCα or CDK4/6 has been demonstrated to impede the nuclear translocation of neutrophil elastase [37]. Furthermore, histone acetylation (e.g., acetylated histone H4) contributes to decondensation by removing the positive charge of histones [44].

The third step is the rupture of the plasma membrane, allowing the release of chromatin extracellularly. Disassembly of the actin cytoskeleton has been demonstrated to weaken the plasma membrane [37]. Furthermore, it has been shown that chromatin decondensation and nuclear swelling provide the physical forces driving the rupture of the nuclear envelope and plasma membrane [37]. The molecular basis for nuclear envelope disintegration is provided by kinase-dependent disassembly of lamin [45]. Nuclear swelling generates physical forces that extend the rupture until the nuclear envelope is completely ruptured. Within the domain of pathology, these molecular mechanisms are identified as prospective therapeutic targets.

### 3.5. PAD6—Regulator of Early Development

PAD6 is the only catalytically inactive isoform, expressed exclusively in oocytes and early embryos [34,46]. It is likely involved in cytoskeletal organization and early developmental processes [47,48].

PAD6 exhibits catalytically inactive behavior due to the absence of preserved Ca^2+^ binding sites and the improper positioning of the catalytic cysteine [46]. Notwithstanding, it performs a pivotal structural function as a constituent of cytoplasmic networks (CPLs) in mammalian oocytes—structures that store maternal proteins, ribosomes, and mRNA necessary for early embryonic development [49]. PAD6 is imperative for the accurate incorporation of ribosomal components into CPLs and the activation of the embryonic genome (EGA) [49,50]. PAD6 knockout in mice and mutations in humans have been demonstrated to cause CPLs to disappear and female infertility by stopping embryo development at the 2-cell stage [50,51]. Furthermore, PAD6 has been demonstrated to be indispensable for the regulation of citrullination in oocytes by virtue of its ability to activate other PAD enzymes, including PAD1, despite the absence of its own enzymatic activity [52].

## 4. New Generation Research Methodologies

### 4.1. Advances in PAD Activity Detection

#### 4.1.1. In Vitro Fluorescence Analysis

Developed in 2023, this method uses a synthetic substrate rich in arginine and a negatively charged dye molecule [53]. It enables the characterization of endogenous PAD activity in complex biological samples, including synovial fluid from patients with arthritis [23]. The method reveals different patterns of citrullination characteristic of specific autoimmune diseases [53].

#### 4.1.2. HPLC-UV Method with Increased Precision

Replacing hydrophobic BAEE with hydrophilic l-arginine as a substrate resulted in a threefold increase in accuracy (IC50 GSK484: 153 nM vs. 527 nM in the COLDER method) [54]. The method provides better separation of reaction products, a lower detection limit (0.5 nmol of citrulline), and a reduced analysis time to 7 min [55].

### 4.2. New Diagnostic Biomarkers

The identification of specific biomarkers significantly expands diagnostic capabilities.

Citrullinated histones in plasma serve as markers of bacterial and viral sepsis [56,57,58,59,60]. ACPA antibodies, which recognize PAD enzyme products (citrullinated proteins), and RF are diagnostic and prognostic biomarkers in rheumatoid arthritis [61,62]. PAD4 in cancer progression, especially in HPV-associated cervical cancer [63,64].

## 5. Pathophysiology of Non-Cancerous Diseases

### 5.1. Autoimmune Diseases

#### 5.1.1. Rheumatoid Arthritis—An Autoimmune Paradigm

RA is a classic example of a disease driven by PAD dysregulation. Of the five isozymes, PAD2 and PAD4 play key roles at the genetic and cellular levels [65]. Citrullination of extracellular matrix proteins (type II collagen, fibrinogen, vimentin) creates neoantigens recognized by autoantibodies (ACPA—anti-citrullinated protein antibodies) [66].

Immune complexes containing citrullinated fibrinogen and anti-citrullinated protein antibodies (ACPA) co-stimulate macrophages via Toll-like receptor 4 (TLR4) and Fcγ receptors, inducing the production of pro-inflammatory cytokines (tumor necrosis factor-alpha [TNF- α] and interleukin-1 beta [IL-1β]) and perpetuating inflammation [67].

Importantly, recent studies show that activation of the Wnt pathway correlates with disease activity in rheumatoid arthritis (RA) and psoriatic arthritis. Elevated DKK1 concentrations in RA positively correlate with the DAS28-CRP index and the bone resorption marker CTX-1. This suggests a synergistic action with PAD-dependent citrullination of type II collagen in the process of joint destruction [68].

PPAD citrullinates human fibrinogen and α-enolase, which are the primary targets of ACPA autoantibodies that are characteristic of RA [69]. The mechanism of action involves the disruption of immune tolerance. PPAD, a bacterial protein, has been shown to induce an immune response against citrullinated peptides. This response subsequently activates the complement system, leading to the release of chemotactic factors C3a and C5a [70,71].

Research conducted on a murine model has demonstrated that a PPAD-deficient strain of *P. gingivalis* results in a substantial reduction in periodontal inflammation, diminished joint swelling, decreased erosive damage, and reduced ACPA levels in comparison to the wild-type strain [72].

#### 5.1.2. Multiple Sclerosis—PAD-Dependent Demyelination

A characteristic pattern of PAD expression is observed in MS: PAD2 is upregulated in glial cells, while PAD4 is expressed in infiltrating immune cells [73]. Citrullination of MBP alters its physical properties, compromising the integrity of the myelin sheath and initiating an autoimmune response against the modified epitopes [22,74].

#### 5.1.3. New Therapeutic Applications

In sepsis, PAD2 deficiency in mouse models increases survival by reducing NETosis and caspase-11-dependent pyroptosis [75,76]. Unlike PAD4, PAD2 deficiency reduces macrophage pyroptosis [77].

In type 1 diabetes, PAD inhibitors may prevent the formation of autoimmune neoepitopes and the destruction of pancreatic β-cells by modulating inflammation prior to pancreatic islet destruction [78].

In Crohn’s disease, however, PAD4 participates in the early development of intestinal fibrosis by activating fibroblasts via the TLR2/NF-κB pathway, where NETs serve as the initial pathological stimulus [79].

### 5.2. Dermatological Diseases

In psoriasis, complex patterns of PAD1, PAD2, and PAD4 dysregulation are observed [80]. Reduced citrullination of filaggrin and keratins (caused by PADI downregulation) impairs epidermal barrier function and promotes inflammation [81]. The correlation between PAD activity and the infiltration of Th17 cells, neutrophils, and dendritic cells indicates a close relationship between citrullination and the immune response [82]. The mechanism of action of PAD-4 in psoriasis involves the initiation of histone citrullination and chromatin decondensation, which is the first step in NET formation [83]. This process leads to the release of protein-DNA complexes into the extracellular space, where NET structures rich in cytoplasmic granules containing neutrophil elastase (NE), myeloperoxidase (MPO), and cathelicidin LL-37 are formed. These proteins not only directly affect keratinocytes but also activate the LL-37/DNA complex, which in turn stimulates plasmacytoid dendritic cells to produce interferon-α and activate the TNFα/IL-17/IL-23 pathway [83]. Serum PAD-4 levels are significantly elevated in patients with psoriasis and correlate with disease severity as assessed by PASI and BSA scales. Furthermore, systemic therapies, particularly anti-TNFα (adalimumab, infliximab), effectively reduced PAD-4 levels after 12 weeks of treatment, with the greatest reduction reaching 81%. This phenomenon may be related to the inhibition of TNFα production at various stages of psoriasis pathogenesis, including NET formation [84].

### 5.3. Porphyromonas Gingivalis in Citrullination and Associated Diseases

Porphyromonas gingivalis is a distinctive bacterium that produces peptidyl arginine deiminase (PPAD), an enzyme that catalyzes the conversion of arginine residues in proteins to citrulline [85]. While the role of P. gingivalis in autoimmune diseases has been discussed previously (Section 5.1.1.), its contribution to other non-cancerous systemic diseases through PPAD activity warrants detailed consideration. PPAD functions in synergy with arginine gingipains (Rgp), which are proteases that cleave proteins after arginine residues, exposing C-terminal arginines. These arginines are then citrullinated by PPAD. In contrast to the preferences exhibited by human PADs, bacterial PPAD demonstrates a distinct predilection for C-terminal arginines [86].

#### 5.3.1. Alzheimer’s Disease

The presence of *P. gingivalis* and its gingipains has been identified in the brains of Alzheimer’s patients, and their levels have been correlated with tau and ubiquitin pathology. The induction of oral infection in mice resulted in brain colonization, augmented Aβ1-42 production, and neurotoxicity [87]. The bacterium disseminates virulence factors to the brain via extracellular membrane vesicles (OMVs), inducing systemic inflammation, leading to cerebrospinal fluid inflammation, and accelerating disease progression [88,89].

#### 5.3.2. Atherosclerosis

*P. gingivalis* and its DNA have been detected in human atherosclerotic plaques. The bacterium has been shown to induce endothelial dysfunction, promote foam cell formation, and stimulate smooth muscle cell proliferation [90]. Additionally, it has been observed to disrupt T cell balance, thereby accelerating the aforementioned processes [91].

Citrullination by PPAD has been demonstrated to disrupt complement system activity by inactivating C5a, facilitate invasion of gingival fibroblasts by inducing prostaglandin E2, and modulate the functions of vasoactive peptides [92].

## 6. PAD in Oncogenesis and Tumor Progression

### 6.1. Oncogenic Mechanisms of PAD2

PAD2 shows selective expression in certain types of cancer, particularly in luminal breast cancer, where it correlates with disease progression [93,94]. Its oncogenic effects include epigenetic regulation—citrullination of histones H3 and H4 leads to chromatin decondensation and changes in gene expression patterns [35,95]. In breast cancer, PAD2 citrullinates the estrogen receptor, promoting the expression of estrogen-dependent genes even at low hormone levels [96].

PAD2 mediates oncogenic processes by modulating EGF signaling [13,97]. Promotion of invasion—citrullination of cytoskeletal proteins (actin, vimentin, tubulin) increases cell migration abilities [95]. PAD2 also participates in epithelial–mesenchymal transition (EMT) [20,75,94].

### 6.2. The Dual Role of PAD4 in Cancer

PAD4 exhibits various functions in cancer biology [98]. Oncogenic functions p53 and PAD4 form a regulatory network in response to DNA damage, where p53 transactivates the expression of PADI4, which then citrullinates regulatory proteins (ING4, NPM1), modulating their functions and subcellular localization, ultimately supporting the tumor-suppressor functions of p53 [99,100]. Activation of GSK3β through citrullination supports antitumor processes through p53 activation and apoptosis induction [101,102].

PAD4 acts as an oncogene by repressing p53 suppressor genes, citrullinating histones H3 and H4 in their promoters, which reduces their expression and promotes cancer cell proliferation. Excessive activity can induce apoptosis by damaging mitochondria [12,103].

### 6.3. The Groundbreaking Discovery of Cellular Extrachromatin Cancer Networks (CECN)

Cellular Extrachromatin Cancer Networks (CECN) are an important finding in cancer biology [104,105]. PAD4, thanks to its constitutive nuclear localization, plays a key role in the formation of extrachromosomal networks [85]. It coordinates the stress response in the tumor microenvironment and facilitates metastasis by preparing cells for invasion [104,105,106,107].

### 6.4. NETs and the Tumor Microenvironment

PAD4-dependent neutrophil networks in tumor tissue constitute a biological platform that both captures dispersed tumor cells and induces their growth and reactivation of dormant tumor foci [108,109]. NETs promote a pro-tumorigenic environment through their dual pathological role: they promote vascular angiogenesis through NFκB activation and support tumor progression through matrix degradation and facilitation of metastasis [110,111]. Recent translational studies have revealed that anti-β2GPI antibodies induce NET formation and directly activate endothelial cells, leading to the expression of tissue factor and adhesion molecules and creating a prothrombotic microenvironment in antiphospholipid syndrome [112]. The detection of elevated NET levels in patients with pulmonary vascular diseases and cancers is clinically relevant, opening up therapeutic opportunities through PAD4 inhibition or NET degradation using DNase I [110,111].

## 7. Therapeutic Advances: New-Generation Inhibitors

### 7.1. Progress in the Design of PAD2 and PAD4 Inhibitors

The years 2019–2025 brought fundamental advances in the development of peptidylarginine deiminase inhibitors, particularly in terms of selectivity and pharmacodynamic properties. One of the most important achievements is the development of JBI-589, an oral PAD4 inhibitor with high selectivity (IC50 = 0.122 μM) and no inhibitory activity against other PAD enzymes even at a concentration of 30 μM [113,114]. JBI-589 has demonstrated efficacy in vivo in mouse models of arthritis, and its oral availability significantly improves patient compliance [114] (Table 2).

The development of BB-Cl-amidine represents a significant advance in the design of PAD4 inhibitors. This compound exhibits more than 20-fold higher cellular activity than Cl-amidine (EC50: 8.8 ± 0.6 μM vs. >200 μM) and significantly improved pharmacokinetics with a half-life of 1.75 h compared to ~15 min for its predecessor. Key structural modifications include the introduction of benzimidazole to prevent proteolysis and a biphenyl residue to increase cellular permeability. In vivo studies in the MRL/lpr model confirmed therapeutic efficacy in reducing NET, improving endothelial function, and protecting against multi-organ damage, opening up new therapeutic perspectives in autoimmune diseases [118].

GSK484, a product of research conducted by GlaxoSmithKline, represents a novel approach in the design of PAD4 inhibitors. This innovative compound is characterized by high selectivity, a reversible mechanism of action, and exhibits an IC50 of 50 nM in the absence of calcium, which increases to 3.2 μM in the presence of physiological concentrations of this cation [118,119]. The mechanism of action manifests by blocking the citrullination of PAD4 target proteins in human neutrophils and inhibiting the formation of neutrophil extracellular traps (NETs) in both mice and humans [119]. The compound exhibits favorable pharmacokinetic profiles with low to moderate clearance and good volume of distribution and half-life in mice and rats, predisposing it for use as a potential therapeutic tool in vivo [120].

In the field of selective inhibition of PAD enzymes, the combination of PAD2 inhibitor (AFM-30a) with PAD4 inhibitor (GSK199) allows for effective blocking of the activity of both isoenzymes without the cytotoxic effects observed for the pan-inhibitor BB-Cl-amidine [121]. Studies have shown that both compounds exhibit minimal toxicity to CD4+, CD8+, B cells, NK cells, and monocytes in the range of 1–20 μM, while BB-Cl-amidine causes cell death at concentrations above 1 μM [121]. This significantly better cellular tolerance, combined with high selectivity of action, suggests a lower risk of adverse effects and greater therapeutic potential compared to non-selective PAD inhibitors [121].

The development of selective PAD2 inhibitors is particularly challenging due to the structural similarities between PAD isoforms. Despite years of effort, the development of isoform-specific inhibitors remains challenging, with major limitations including limited efficacy in cell tests and the lack of clinically approved selective PAD2 inhibitors [122].

#### The Clinical Status and Translational Prospects of the Subject Are as Follows

Despite two decades of intensive research on peroxisome proliferator-activated receptor (PAP) inhibitors, as of the present moment (2024–2025), no PAP inhibitor has been approved for clinical use in humans. This discrepancy between the initial optimism instigated by the preliminary findings and the subsequent absence of clinical translation is indicative of the intricacies inherent to this particular class of targets [116,123]. Among the extensive array of inhibitors that have been developed, only two programs have attained advanced stages of development. JBI-589, developed by Jubilant Therapeutics, is a selective, orally bioavailable, and potent inhibitor of PAD4. In preclinical models of CIA in mice, JBI-589 has demonstrated significant efficacy, as reported in 2023. The compound is currently undergoing preclinical trials with potential applications in autoimmune diseases and cancer. However, no PAD4 inhibitor has yet been approved for clinical use [114,115]. (2) Anti-PAD4 monoclonal antibodies (Bristol-Myers Squibb/AstraZeneca) are being prepared for clinical trials for RA, including AZD1163, a bispecific anti-PAD2/4 antibody in phase I trials (NCT06103877) [124,125]. BMS’s strategic shift from small molecules (following the synthesis of over 3500 compounds) to biologics indicates a reevaluation of the efficacy of the macromolecular approach in achieving clinical success [116].

The primary translational challenges encompass the following: The issue of selectivity is a primary concern, as early pan-PAD inhibitors (Cl-amidine, BB-Cl-amidine) carry the risk of adverse effects resulting from the inhibition of physiologically important isozymes [2,22]. Additionally, pharmacological limitations are evident in the short half-lives of these early inhibitors (Cl-amidine: ~15 min; BB-Cl-amidine: ~1.75 h), which lead to a decrease in cellular versus biochemical potency (~10×) and a Ca^2+^ concentration dependence for the GSK series (GSK199: 200 nM without Ca^2+^ vs. 1000 nM with 2 mM Ca^2+^; GSK484: 50 nM without Ca^2+^ vs. 250 nM with 2 mM Ca^2+^) [120,121]. Thirdly, there is uncertainty regarding the long-term safety of citrullination. Citrullination is physiologically important in a variety of processes, including keratinization, myelin stability, and NET formation. Furthermore, PAD2 and PAD4 are expressed in both cancerous and normal cells [119].

Fourthly, the complexity of diseases is a result of studies on IBD, in which GSK484 reduced NET formation but did not improve clinical parameters, such as the disease activity index, intestinal histoarchitecture damage, and inflammatory markers. This suggests that PAD4 monotherapy may be insufficient [126,127].

The forthcoming years will reveal whether PAD4 inhibitors will be utilized clinically. Presently, the most advanced small molecule inhibitor is JBI-1044 (Jubilant Therapeutics), a potentially optimized version of the previously described JBI-589, which is currently in the IND-enabling stage. Concurrently, BMS is developing an alternative approach based on anti-PAD4 antibodies [113,116].

### 7.2. Therapeutic Applications in Oncology

Clinical applications of novel PAD inhibitors span a wide spectrum of cancers. In colorectal cancer, GSK484 promotes radiosensitivity of cells by inducing double-strand DNA breaks and inhibits NET formation in vivo [128]. In triple-negative breast cancer, pretreatment with GSK484 enhances radiation-induced effects of inhibiting cell proliferation, migration, and invasion, while facilitating apoptosis [128].

### 7.3. Targeting CECN in Immunotherapy

Current therapeutic strategies focus on the use of selective inhibitors such as JBI-589 and GSK484 to inhibit tumor progression by reducing CXCR2 expression and blocking neutrophil chemotaxis. Targeting PAD4 in tumor-associated macrophages is particularly promising, as PAD4 inhibition negatively correlates with clinical response to immune checkpoint blockade therapy [64,115,129].

The strategy of targeting CECN (chromatin extracellular chromatin networks) through PAD4 inhibition may restore the anti-tumorogenic functions of macrophages and improve CD8+ T cell infiltration in the tumor microenvironment, which is a promising direction for combination therapies with immunotherapy [115,129,130].

### 7.4. Limitations and Translational Challenges

Despite significant progress, important translational challenges remain. The main limitation of GSK484 is uncertainty regarding its functionality in vivo due to the requirement for low calcium concentrations for optimal binding to the target, which may be difficult to achieve under physiological conditions [64,119]. Additionally, a 2023 study showed that GSK484 induces phospholipidosis at the recommended concentration of 10 μM, indicating potential adverse effects of this chemical probe at high concentrations [131].

Overall challenges in the development of PAD inhibitors include limited efficacy in cell-based assays and the lack of clinically approved selective PAD2 inhibitors. Although reversible PAD inhibitors such as paclitaxel, minocycline, and streptomycin exist, their efficacy remains suboptimal, particularly for halogenated acetamidine-based inhibitors [114,132,133].

The future of PAD4 and PAD2 inhibition-based therapies will require further development of more selective compounds with improved pharmacokinetic properties, advanced patient stratification strategies, and innovative clinical trial designs [115]. The increase in response rates to therapies using biomarkers demonstrates the potential of this strategy, but the full realization of the therapeutic potential of PAD enzymes in precision medicine requires overcoming current limitations in selectivity, cellular efficacy, and translation to clinical applications [134].

## 8. Clinical Prospects and Challenges

The latest clinical prospects for PAD4 and PAD2 enzymes are currently one of the most promising areas of development for targeted therapies in oncology and autoimmune diseases. Advances in understanding the mechanisms of protein citrullination catalyzed by peptidylarginine deiminase open up new therapeutic possibilities, while revealing significant challenges related to selectivity, cellular efficacy, and clinical trial design.

### 8.1. New Therapeutic Strategies

Inhibition of PAD4 in tumor-associated macrophages (TAM) is a particularly promising therapeutic approach, as PAD4 negatively correlates with clinical response to immune checkpoint blockade therapy. This mechanism involves citrullination of STAT1 at arginine 121, leading to enhanced STAT1-PIAS1 interaction and loss of STAT1 transcriptional activity, ultimately limiting MHC class II expression [130].

PAD4 inhibitors show significant potential in immunomodulation, particularly in the context of combination therapies with immunotherapy. Selective inhibitors such as JBI-589 and GSK484 are currently being developed to inhibit tumor progression by reducing CXCR2 expression and blocking neutrophil chemotaxis [114]. The precision medicine strategy in this context focuses on identifying patients with high PAD4 expression in tumor tissues as potential beneficiaries of PAD inhibitor therapy [64].

### 8.2. Companion Biomarkers

Histone H3 citrullination (H3cit) is considered the most specific biomarker of circulating NETs (neutrophil extracellular traps). Studies indicate that elevated plasma H3cit levels are associated with a twofold increase in the risk of short-term mortality in patients with advanced cancer [135,136]. This biomarker shows a high correlation with clinical burden and prognostic outcomes, making it a potential diagnostic and predictive tool [137,138].

PAD2 also shows potential as a biomarker in sepsis, where its concentration is associated with lactate and procalcitonin levels [75,137]. Monitoring PAD2 activity may serve as an indicator of treatment progress in inflammatory and autoimmune diseases [75]. In the context of precision medicine, PAD2 shows different expression patterns depending on the type of cancer—it is high in tumor tissues and blood of patients with breast, liver, stomach, and cervical cancer [138,139], but appears to act as a tumor suppressor in colorectal cancer [140].

### 8.3. Limitations and Challenges

The main problems in the field of PAD inhibitors are the insufficient efficacy of existing compounds in cellular studies and the lack of selective PAD2 inhibitors approved for clinical use. Currently available reversible inhibitors (paclitaxel, minocycline, streptomycin) show limited activity, especially those based on halogenated acetamidine derivatives [123].

Despite years of effort, the development of isoform-specific inhibitors remains a challenge. Selectivity is a critical issue—JBI-589 exhibits high selectivity for PAD4 with an IC50 of 0.122 μM, but shows no inhibitory activity against PAD1, PAD2, PAD3, and PAD6 even at the highest concentrations tested [113,114].

Methods for testing PAD activity encounter difficulties related to background interference from colored plant extracts, which limits the effectiveness of traditional methods based on color development or fluorescence. The mechanism of PAD4 activation during NETosis in vivo remains unclear, including the required calcium concentration and the effect of calcium production on the PAD4 activation mechanism [141].

In clinical trials, the main challenge remains the heterogeneity of tumors and the complexity of immunotherapeutic resistance mechanisms. More recent biomarker-based clinical trials have shown improved response rates, progression-free survival, and overall survival compared to clinical trials that did not use a biomarker for patient selection [142]. Nevertheless, analysis of FDA approvals indicates that PD-L1 was predictive in only 28.9% of cases, highlighting the limitations of single biomarkers [30].

The future of PAD4- and PAD2-based therapies will require the development of more selective and safer inhibitors with improved bioavailability, advanced strategies for identifying biomarkers such as H3Cit for patient stratification, and the use of innovative clinical trial platforms utilizing master protocols to optimize therapy selection [130]. Growing evidence for the role of PAD enzymes in tumor progression, NET formation, and metastatic processes points to the significant therapeutic potential of this strategy, particularly in the context of combination therapies with immunotherapy [64]. Further research into the mechanisms of resistance and the identification of patient populations who will benefit most from anti-PAD therapy is crucial to fully exploit the potential of this therapeutic pathway in precision oncology [117].

## 9. Future Prospects

### 9.1. Evolution of the Paradigm

The last five years have fundamentally changed the perception of PAD enzymes—from rare post-translational modifiers to key regulators of cellular processes [143,144]. Citrullination is now recognized as an important regulatory mechanism comparable to phosphorylation or ubiquitination [4,80].

### 9.2. Emerging Concepts

Reversible inhibitors: An alternative to irreversible haloacetamide compounds with better control of enzymatic activity [145,146].

Combination strategies: Combining PAD inhibitors with other targeted therapies to exploit synergistic mechanisms of action [115].

Drug repositioning: Some strategies approved for autoimmune diseases may be effective in various indications [53,147].

### 9.3. Future Directions for Research

- The precise mechanisms of CECN regulation and their role in cancer heterogeneity [130].
- Development of predictive biomarkers for personalized therapy [24].
- Research on enzymatic vs. non-enzymatic PAD function [32,148].
- The role of PAD in aging and neurodegenerative diseases [7,20].

## 10. Conclusions

PAD enzymes are a fascinating family of proteins of fundamental importance for cellular homeostasis and disease pathogenesis. The last five years have brought substantial advances that have expanded our understanding of their biological functions and therapeutic potential.

### 10.1. Key Achievements Include

1. Revision of localization mechanisms—discovery of regulated nuclear translocation of PAD2 independent of the classical NLS.
2. Identification of CECNs—extrachromosomal cancer networks—as a new mechanism of intercellular communication.
3. Development of a new generation of inhibitors with improved selectivity, stability, and safety profile.
4. Advances in diagnostic methods enabling precise monitoring of PAD activity.
5. Expansion of the spectrum of clinical applications from autoimmune diseases to oncology.

### 10.2. Translational Prospects

Intensive industrial development with numerous patents and innovative drug delivery systems indicate the high commercialization potential of anti-PAD therapies. The combination of advances in understanding molecular mechanisms, the development of selective inhibitors, and targeted delivery systems provides tools for precise modulation of PAD activity in various disease contexts.

### 10.3. Future Challenges

Despite impressive progress, many questions remain unanswered. Optimizing inhibitor selectivity, developing companion biomarkers, and combination strategies will be key to maximizing therapeutic benefits while minimizing side effects.

The era of PAD enzymes as therapeutic targets is just beginning. Their full potential in personalized medicine, precision oncology, and autoimmune disease therapy will be revealed in the coming years, promising new treatment opportunities for millions of patients worldwide.

## Figures and Tables

**Table 1:** Functional characteristics of PAD isozymes.

Isozyme	Tissue Localization	Physiological Functions	Key Substrates	Related Diseases
PAD1	Epidermis, sweat glands	Keratinocyte differentiation, keratosis	Filaggrin, keratins	Psoriasis, keratinization disorders
PAD2	CNS, skeletal muscles, immune cells	Myelin plasticity, gene regulation, METosis	MBP, histones H3/H4, vimentin	MS, ALS, sepsis, breast cancer
PAD3	Skin, hair follicles	Skin barrier integrity	Filaggrin, keratins	Alopecia, hair diseases
PAD4	Neutrophils, hematopoietic cells	Histone citrullination, NETosis, CECN	Histones H3/H4, nuclear proteins	RA, lupus, cancer, sepsis
PAD6	Oocytes, early embryos	Oocyte cytoskeletal organization	Unknown	Infertility, developmental defects

**Table 2:** Comparative characteristics of PAD inhibitors.

Inhibitor	Type	Select	IC50	PK (t½)	Key Advantages	Key Disadvantages	Stage	Ref.
Cl-amidine	Irrevers.	Pan-PAD	5.9 μM (PAD2)	15 min	Preclinical efficacy	Low potency, short t½	Research tool	[115]
BB-Cl-amidine	Irrevers.	Pan-PAD	8.8 μM (cells)	1.75 h	10× potency vs. Cl-amid.	No selectivity, toxicity	Preclinical	[115]
TDFA	Irrevers.	PAD4	2.3 μM	ND	15× PAD4 selectivity	Irreversible, peptide-like	Research tool	[115]
GSK484	Revers.	PAD4	50 nM	ND	High potency, selectivity	Ca^2+^ dependence, GSK→mAb	Research tool	[115]
GSK199	Revers.	PAD4	200 nM	ND	PAD4 selectivity	Lower potency	Research tool	[115]
JBI-589	Revers.	PAD4	0.12 μM	ND	Oral, CIA efficacy	Rapid metabolism	Preclinical	[115]
JBI-1044	Revers.	PAD4	ND	ND	JBI-589 successor	Limited data	★ IND-enabling ★	[116,117]
mAb (BMS)	Biologic	PAD4	ND	Unpublished pharmacokinetic data	Long t½, selectivity	i.v./s.c. admin., cost	Phase I preparation	[116,117]

Abbreviations: Selectivity = select; PK = pharmacokinetics; t½ = half-life; cells = cells; ND = no data; mAb = monoclonal antibodies; BMS = Bristol-Myers Squibb; i.v. = intravenous; s.c. = subcutaneous. ★ JBI-1044 and BMS mAb = closest to clinical trials.

## Data Availability

No new data were created or analyzed in this study. Data sharing is not applicable to this article.
